# Supplementary material for: Bioactive compounds, antioxidant capacity and anti-inflammatory activity of native fruits from Brazil
Source: PLoS One. 2023 May 10;18(5):e0285625. doi: 10.1371/journal.pone.0285625 (PMC10171607; doi:10.1371/journal.pone.0285625)
Supplement: Attachment — Submitted filename: Response to Reviewers.docx [file pone.0285625.s002.docx]

# Food Science

2510 River Drive

Knoxville, TN 37996

Office: 865-974-7331

# foodscience.tennessee.edu

January 4, 2023

Umakanta Sarker
Academic Editor
PLOS ONE

Dear Dr. Sarker,

Thank you very much for orchestrating the review of our manuscript entitled “Bioactive compounds, antioxidant capacity and anti-inflammatory activity of native fruits from Brazil” for potential publication in PLOS One. This study involved several Brazilian native fruit producers, fruit researchers at the Federal University of Rio Grande do Sul, Brazil and the University of Tennessee, United States. These native fruits have great potential for application as a source of bioactive compounds with very promising antioxidant and anti-inflammatory properties. It is known that there is a great diversity of native fruits in Brazil, however there is a lack of more advanced studies in the area of detailing the chemical composition and pharmacological activities, and these are extremely important to stimulate the production chain in this sector, so that greater investment occurs in the area and also application of these vegetable sources in food products.

Our study aims to disseminate these results for grumixama and guabiju fruits, of which there is little material published in the literature. We believe that with these results we will be able to help stimulate the production, consumption, and production chain of native Brazilian fruits.

Below, you will find the point-by-point response to the reviews and suggestions made by the reviewers and PLOS One editorial team. The minimal set has been uploaded as Supplementary Material file. We are also uploading both marked and unmarked version of the manuscript.

Thank you very much for considering this work for publication.

Sincerely,

VERMONT P DIA, Ph D

Associate Professor

Department of Food Science

The University of Tennessee

2510 River Dr Knoxville TN 37996

[vdia@utk.edu](mailto:vdia@utk.edu), 865-974-7265

Journal Requirements:

When submitting your revision, we need you to address these additional requirements.

1. Please ensure that your manuscript meets PLOS ONE's style requirements, including those for file naming. The PLOS ONE style templates can be found at

[https://journals.plos.org/plosone/s/file?id=wjVg/PLOSOne_formatting_sample_main_body.pdf](https://nam11.safelinks.protection.outlook.com/?url=https%3A%2F%2Fjournals.plos.org%2Fplosone%2Fs%2Ffile%3Fid%3DwjVg%2FPLOSOne_formatting_sample_main_body.pdf&data=05%7C01%7Cvdia%40utk.edu%7Cfb0c2252d66543d3b1f408dad96c145e%7C515813d9717d45dd9eca9aa19c09d6f9%7C0%7C0%7C638061355584103967%7CUnknown%7CTWFpbGZsb3d8eyJWIjoiMC4wLjAwMDAiLCJQIjoiV2luMzIiLCJBTiI6Ik1haWwiLCJXVCI6Mn0%3D%7C3000%7C%7C%7C&sdata=Xrfte4s8OrmPuK8WyxfA4FgZjELsPGrr56anK37rl%2Fg%3D&reserved=0)  and

[https://journals.plos.org/plosone/s/file?id=ba62/PLOSOne_formatting_sample_title_authors_affiliations.pdf](https://nam11.safelinks.protection.outlook.com/?url=https%3A%2F%2Fjournals.plos.org%2Fplosone%2Fs%2Ffile%3Fid%3Dba62%2FPLOSOne_formatting_sample_title_authors_affiliations.pdf&data=05%7C01%7Cvdia%40utk.edu%7Cfb0c2252d66543d3b1f408dad96c145e%7C515813d9717d45dd9eca9aa19c09d6f9%7C0%7C0%7C638061355584103967%7CUnknown%7CTWFpbGZsb3d8eyJWIjoiMC4wLjAwMDAiLCJQIjoiV2luMzIiLCJBTiI6Ik1haWwiLCJXVCI6Mn0%3D%7C3000%7C%7C%7C&sdata=8HhDZwHDSkbPAz%2FBsBR4GMxoAdJoFKViXbCuujOwihw%3D&reserved=0)

**Response.** The manuscript has been formatted according to PLOS ONE’s style requirements.

2. We note that the grant information you provided in the ‘Funding Information’ and ‘Financial Disclosure’ sections do not match.

When you resubmit, please ensure that you provide the correct grant numbers for the awards you received for your study in the ‘Funding Information’ section.

**Response.** This has been updated.

3. Thank you for stating the following financial disclosure:

“No”

At this time, please address the following queries:

a)        Please clarify the sources of funding (financial or material support) for your study. List the grants or organizations that supported your study, including funding received from your institution.

b)        State what role the funders took in the study. If the funders had no role in your study, please state: “The funders had no role in study design, data collection and analysis, decision to publish, or preparation of the manuscript.”

c)        If any authors received a salary from any of your funders, please state which authors and which funders.

d)        If you did not receive any funding for this study, please state: “The authors received no specific funding for this work.”

Please include your amended statements within your cover letter; we will change the online submission form on your behalf.

**Response.** Done.

4.  In your Data Availability statement, you have not specified where the minimal data set underlying the results described in your manuscript can be found. PLOS defines a study's minimal data set as the underlying data used to reach the conclusions drawn in the manuscript and any additional data required to replicate the reported study findings in their entirety. All PLOS journals require that the minimal data set be made fully available. For more information about our data policy, please see <http://journals.plos.org/plosone/s/data-availability>.

Upon re-submitting your revised manuscript, please upload your study’s minimal underlying data set as either Supporting Information files or to a stable, public repository and include the relevant URLs, DOIs, or accession numbers within your revised cover letter. For a list of acceptable repositories, please see <http://journals.plos.org/plosone/s/data-availability#loc-recommended-repositories>. Any potentially identifying patient information must be fully anonymized.

Important: If there are ethical or legal restrictions to sharing your data publicly, please explain these restrictions in detail. Please see our guidelines for more information on what we consider unacceptable restrictions to publicly sharing data: <http://journals.plos.org/plosone/s/data-availability#loc-unacceptable-data-access-restrictions>. Note that it is not acceptable for the authors to be the sole named individuals responsible for ensuring data access.

We will update your Data Availability statement to reflect the information you provide in your cover letter.

**Response.** The minimal data set has been uploaded as Supporting Information file.

5. Please upload a new copy of Figure 1 as the detail is not clear. Please follow the link for more information: <https://blogs.plos.org/plos/2019/06/looking-good-tips-for-creating-your-plos-figures-graphics/>" [https://blogs.plos.org/plos/2019/06/looking-good-tips-for-creating-your-plos-figures-graphics/](https://nam11.safelinks.protection.outlook.com/?url=https%3A%2F%2Fblogs.plos.org%2Fplos%2F2019%2F06%2Flooking-good-tips-for-creating-your-plos-figures-graphics%2F&data=05%7C01%7Cvdia%40utk.edu%7Cfb0c2252d66543d3b1f408dad96c145e%7C515813d9717d45dd9eca9aa19c09d6f9%7C0%7C0%7C638061355584103967%7CUnknown%7CTWFpbGZsb3d8eyJWIjoiMC4wLjAwMDAiLCJQIjoiV2luMzIiLCJBTiI6Ik1haWwiLCJXVCI6Mn0%3D%7C3000%7C%7C%7C&sdata=9GJbAHORm0lDIEONPNBzEtcumth1WfuwWH7AA3RIjaI%3D&reserved=0)

[Note: HTML markup is below. Please do not edit.]

**Response.** This has been re-uploaded with better quality and higher resolution.

Reviewers' comments:

Reviewer's Responses to Questions

**Comments to the Author**

1. Is the manuscript technically sound, and do the data support the conclusions?

The manuscript must describe a technically sound piece of scientific research with data that supports the conclusions. Experiments must have been conducted rigorously, with appropriate controls, replication, and sample sizes. The conclusions must be drawn appropriately based on the data presented.

Reviewer #1: Yes

Reviewer #2: Yes

2. Has the statistical analysis been performed appropriately and rigorously?

Reviewer #1: Yes

Reviewer #2: Yes

3. Have the authors made all data underlying the findings in their manuscript fully available?

The [PLOS Data policy](https://nam11.safelinks.protection.outlook.com/?url=http%3A%2F%2Fwww.plosone.org%2Fstatic%2Fpolicies.action%23sharing&data=05%7C01%7Cvdia%40utk.edu%7Cfb0c2252d66543d3b1f408dad96c145e%7C515813d9717d45dd9eca9aa19c09d6f9%7C0%7C0%7C638061355584103967%7CUnknown%7CTWFpbGZsb3d8eyJWIjoiMC4wLjAwMDAiLCJQIjoiV2luMzIiLCJBTiI6Ik1haWwiLCJXVCI6Mn0%3D%7C3000%7C%7C%7C&sdata=BB1VRMFqC88hgZ0MiOtYnT%2FOR5vxV1FcjsYhci%2FY6mc%3D&reserved=0) requires authors to make all data underlying the findings described in their manuscript fully available without restriction, with rare exception (please refer to the Data Availability Statement in the manuscript PDF file). The data should be provided as part of the manuscript or its supporting information, or deposited to a public repository. For example, in addition to summary statistics, the data points behind means, medians and variance measures should be available. If there are restrictions on publicly sharing data—e.g. participant privacy or use of data from a third party—those must be specified.

Reviewer #1: Yes

Reviewer #2: Yes

4. Is the manuscript presented in an intelligible fashion and written in standard English?

PLOS ONE does not copyedit accepted manuscripts, so the language in submitted articles must be clear, correct, and unambiguous. Any typographical or grammatical errors should be corrected at revision, so please note any specific errors here.

Reviewer #1: Yes

Reviewer #2: Yes

5. Review Comments to the Author

Please use the space provided to explain your answers to the questions above. You may also include additional comments for the author, including concerns about dual publication, research ethics, or publication ethics. (Please upload your review as an attachment if it exceeds 20,000 characters)

Reviewer #1: 1. In Abstract, kindly put the values from the results into the abstract and also show the comparison, abstract should be re-write.

**Response.** The Abstract has been re-written per reviewer’s recommendations. Thank you.

2. In Introduction, authors must declassify the native fruit names (including common and scientific names).

**Response.** This has been highlighted in page 3, lines 68-70.

3. In Materials and Methods section, in DPPH method reference is missing.

**Response.** This is reference 18 as highlighted in pages 7-8, lines 173-176.

4. Conclusion should be re-write.

**Response.** This has been re-written to give a broad and general conclusion of the findings without repeating the results section.

5. Figure 1 and 3 quaility is too poor.

**Response.** These figures were modified and re-uploaded with better quality and high resolution.

Reviewer #2: This manuscript has reasonable data to support the conclusions. However, there are some issues that should be clarified or revised as follows:
1. In the introduction part, the reasons should be added for the selection of various standard substances for HPLC quantitative analysis.

**Response.** The following sentences were added in the Introduction. Thank you.

Phenolic compounds were both quantified spectrophotometrically and individual compounds were identified and quantified by high performance liquid chromatography (HPLC) using different analytical standards. These standards were chosen based on availability as well as those reported in the literature to be present in these fruits.

2. In the result part, the results of radicals scavenging and anti-inflammatory effects should be presented in the form of percent inhibition for easier comparison with other studies.

**Response.** We respectfully take this comment into consideration. We believe that the current presentation of results as actual concentrations of each radicals (DPPH and NO) and actual concentrations of inflammatory markers (NO and IL-6) are better since we are using different concentrations of the extracts. Moreover, these are clearly delineated in Table 3 footnote (DPPH and NO) and Figure 3 legend (NO and IL-6) as relative to blank (100%) and positive control (LPS+), respectively. Thank you.

3. In the discussion part:
(1) The authors should detail how the differences of three methods for testing antioxidant activity performed in this study in terms of antioxidant mechanisms were.

**Response.** The following sentences were added in the Discussion section. Thank you.

We used three different *in vitro* assays to determine the antioxidant activity of grumixama and guabiju acidic extracts. DPPH scavenging activity measured the reducing ability of the extracts based mainly on the electron transfer reaction, and considered rapid, simple, and highly sensitive [34]. NO scavenging is based on the spontaneous generation of NO by sodium nitroprusside in aqueous solution upon interaction with oxygen and in the presence of the extracts that competes with oxygen resulted in the reduced production of NO [35]. On the other hand, ORAC assay is a hydrogen atom transfer-based assay where the generated peroxyl radical is measured which can provide the ability of the antioxidant extracts to break radical chain formation [36].

The following references were added:

34. Moon, J. K., & Shibamoto, T. Antioxidant assays for plant and food components. Journal of Agricultural and Food Chemistry, 2009, 57, 1655–1666.

35. Sarwar, R., Farooq, U., Khan, A., Naz, S., Khan, S., Khan, A., Rauf, A., Bahadar, H., & Uddin, R. Evaluation of antioxidant, free radical scavenging, and antimicrobial activity of *Quercus incana* Roxb. Frontiers in Pharmacology, 2015, 6, 277.

36. Huang, D., Ou, B., & Prior, R. L. The chemistry behind antioxidant capacity assays. Journal of Agricultural and Food Chemistry,

2005, 53, 1841–1856.

(2) In order to compare the concentrations of active substances in both plants from this study with those found in other plants in the previous studies, the variation of the extraction methods and testing conditions must take into account.

**Response.** The following sentences were added in the Discussion section. Thank you.

In addition, slight variations in the results found when compared from previous studies can be attributed to differences in extraction protocols (such as time used, reagents, and equipment efficiency), sample storage protocols, and also to differences in analysis parameters and methodology used.

4. The typing errors should be corrected throughout the manuscript.

**Response.** The manuscript has been proof-read to correct typing errors.

5. The image quality in the manuscript should be improved.

**Response.** The images were re-uploaded with better quality and higher resolution.

6. PLOS authors have the option to publish the peer review history of their article ([what does this mean?](https://nam11.safelinks.protection.outlook.com/?url=https%3A%2F%2Fjournals.plos.org%2Fplosone%2Fs%2Feditorial-and-peer-review-process%23loc-peer-review-history&data=05%7C01%7Cvdia%40utk.edu%7Cfb0c2252d66543d3b1f408dad96c145e%7C515813d9717d45dd9eca9aa19c09d6f9%7C0%7C0%7C638061355584260202%7CUnknown%7CTWFpbGZsb3d8eyJWIjoiMC4wLjAwMDAiLCJQIjoiV2luMzIiLCJBTiI6Ik1haWwiLCJXVCI6Mn0%3D%7C3000%7C%7C%7C&sdata=GKPiuMBKaw51%2FQyhUeWg%2Fp3bZF2Gkhvd1Uqof%2BvbUUQ%3D&reserved=0)). If published, this will include your full peer review and any attached files.


If you choose “no”, your identity will remain anonymous but your review may still be made public.


**Do you want your identity to be public for this peer review?** For information about this choice, including consent withdrawal, please see our [Privacy Policy](https://nam11.safelinks.protection.outlook.com/?url=https%3A%2F%2Fwww.plos.org%2Fprivacy-policy&data=05%7C01%7Cvdia%40utk.edu%7Cfb0c2252d66543d3b1f408dad96c145e%7C515813d9717d45dd9eca9aa19c09d6f9%7C0%7C0%7C638061355584260202%7CUnknown%7CTWFpbGZsb3d8eyJWIjoiMC4wLjAwMDAiLCJQIjoiV2luMzIiLCJBTiI6Ik1haWwiLCJXVCI6Mn0%3D%7C3000%7C%7C%7C&sdata=gK4KXSFVC%2FqsqPG1j%2BJMcgH99mCmEfpfl0Q%2B4gLSmT0%3D&reserved=0).

Reviewer #1: No

Reviewer #2: No
