# Supplementary material for: Bioactive compounds, antioxidant capacity and anti-inflammatory activity of native fruits from Brazil
Source: PLoS One. 2023 May 10;18(5):e0285625. doi: 10.1371/journal.pone.0285625 (PMC10171607; doi:10.1371/journal.pone.0285625)
Supplement: Attachment — Submitted filename: Response to Reviewers_R2.docx [file pone.0285625.s003.docx]

# Food Science

2510 River Drive

Knoxville, TN 37996

Office: 865-974-7331

# foodscience.tennessee.edu

April 3, 2023

Umakanta Sarker
Academic Editor
PLOS ONE

Dear Dr. Sarker,

Thank you very much for orchestrating the review of our manuscript entitled “Bioactive compounds, antioxidant capacity and anti-inflammatory activity of native fruits from Brazil” for potential publication in PLOS One. This study involved several Brazilian native fruit producers, fruit researchers at the Federal University of Rio Grande do Sul, Brazil and the University of Tennessee, United States. These native fruits have great potential for application as a source of bioactive compounds with very promising antioxidant and anti-inflammatory properties. It is known that there is a great diversity of native fruits in Brazil, however there is a lack of more advanced studies in the area of detailing the chemical composition and pharmacological activities, and these are extremely important to stimulate the production chain in this sector, so that greater investment occurs in the area and also application of these vegetable sources in food products.

Our study aims to disseminate these results for grumixama and guabiju fruits, of which there is little material published in the literature. We believe that with these results we will be able to help stimulate the production, consumption, and production chain of native Brazilian fruits.

The figures were uploaded and checked by PACE. Below, you will find the point-by-point response to the reviews and suggestions made by the reviewers and PLOS One editorial team. The minimal set has been uploaded as Supplementary Material file. We are also uploading both marked and unmarked version of the manuscript.

Thank you very much for considering this work for publication.

Sincerely,

VERMONT P DIA, Ph D

Associate Professor

Department of Food Science

The University of Tennessee

2510 River Dr Knoxville TN 37996

[vdia@utk.edu](mailto:vdia@utk.edu), 865-974-7265

Journal Requirements:

Please review your reference list to ensure that it is complete and correct. If you have cited papers that have been retracted, please include the rationale for doing so in the manuscript text, or remove these references and replace them with relevant current references. Any changes to the reference list should be mentioned in the rebuttal letter that accompanies your revised manuscript. If you need to cite a retracted article, indicate the article’s retracted status in the References list and also include a citation and full reference for the retraction notice.

**Response.** The reference list has been checked. None of the cited papers was retracted.


[Note: HTML markup is below. Please do not edit.]

Reviewers' comments:

Reviewer's Responses to Questions

**Comments to the Author**

1. If the authors have adequately addressed your comments raised in a previous round of review and you feel that this manuscript is now acceptable for publication, you may indicate that here to bypass the “Comments to the Author” section, enter your conflict of interest statement in the “Confidential to Editor” section, and submit your "Accept" recommendation.

Reviewer #1: All comments have been addressed

Reviewer #3: All comments have been addressed

**Response.** Thank you very much.

2. Is the manuscript technically sound, and do the data support the conclusions?

The manuscript must describe a technically sound piece of scientific research with data that supports the conclusions. Experiments must have been conducted rigorously, with appropriate controls, replication, and sample sizes. The conclusions must be drawn appropriately based on the data presented.

Reviewer #1: Partly

**Response.** The conclusion has been revised per suggestion in Review Comments to the Author.

Reviewer #3: Yes

**Response.** Thank you very much.

3. Has the statistical analysis been performed appropriately and rigorously?

Reviewer #1: Yes

Reviewer #3: Yes

**Response.** Thank you very much.

4. Have the authors made all data underlying the findings in their manuscript fully available?

The [PLOS Data policy](https://nam11.safelinks.protection.outlook.com/?url=http%3A%2F%2Fwww.plosone.org%2Fstatic%2Fpolicies.action%23sharing&data=05%7C01%7Cvdia%40utk.edu%7C7f95ea4e9e3f4595a22808db34665e1f%7C515813d9717d45dd9eca9aa19c09d6f9%7C0%7C0%7C638161386490610613%7CUnknown%7CTWFpbGZsb3d8eyJWIjoiMC4wLjAwMDAiLCJQIjoiV2luMzIiLCJBTiI6Ik1haWwiLCJXVCI6Mn0%3D%7C3000%7C%7C%7C&sdata=Lt2YFz4UHg50fEEmcIYOaR1jimIK%2FMS9IrBoGxeGUKY%3D&reserved=0) requires authors to make all data underlying the findings described in their manuscript fully available without restriction, with rare exception (please refer to the Data Availability Statement in the manuscript PDF file). The data should be provided as part of the manuscript or its supporting information, or deposited to a public repository. For example, in addition to summary statistics, the data points behind means, medians and variance measures should be available. If there are restrictions on publicly sharing data—e.g. participant privacy or use of data from a third party—those must be specified.

Reviewer #1: Yes

Reviewer #3: Yes

**Response.** Thank you very much.

5. Is the manuscript presented in an intelligible fashion and written in standard English?

PLOS ONE does not copyedit accepted manuscripts, so the language in submitted articles must be clear, correct, and unambiguous. Any typographical or grammatical errors should be corrected at revision, so please note any specific errors here.

Reviewer #1: Yes

Reviewer #3: Yes

**Response.** Thank you very much.

6. Review Comments to the Author

Please use the space provided to explain your answers to the questions above. You may also include additional comments for the author, including concerns about dual publication, research ethics, or publication ethics. (Please upload your review as an attachment if it exceeds 20,000 characters)

Reviewer #1: Please make conclusion more scientific as per results obtained. It is not a review but research, so pay attention.

**Response.** The conclusion has been revised according to the results obtained and read as follows:

Our study showed that phenolic compounds from grumixama and guabiju possess antioxidant and anti-inflammatory properties. The results can be used to promote the consumption of these fruits and highlight the potential health benefits of grumixama and guabiju.

Reviewer #3: the revised version reports all the corrections required by the Reviewers. Now the paper can be accepted

**Response.** Thank you very much.

7. PLOS authors have the option to publish the peer review history of their article ([what does this mean?](https://nam11.safelinks.protection.outlook.com/?url=https%3A%2F%2Fjournals.plos.org%2Fplosone%2Fs%2Feditorial-and-peer-review-process%23loc-peer-review-history&data=05%7C01%7Cvdia%40utk.edu%7C7f95ea4e9e3f4595a22808db34665e1f%7C515813d9717d45dd9eca9aa19c09d6f9%7C0%7C0%7C638161386490766846%7CUnknown%7CTWFpbGZsb3d8eyJWIjoiMC4wLjAwMDAiLCJQIjoiV2luMzIiLCJBTiI6Ik1haWwiLCJXVCI6Mn0%3D%7C3000%7C%7C%7C&sdata=MBH%2B5YP7y5WnCwg46nh52eqCeZl9eLTpa4NmF3uhRpA%3D&reserved=0)). If published, this will include your full peer review and any attached files.


If you choose “no”, your identity will remain anonymous but your review may still be made public.


**Do you want your identity to be public for this peer review?** For information about this choice, including consent withdrawal, please see our [Privacy Policy](https://nam11.safelinks.protection.outlook.com/?url=https%3A%2F%2Fwww.plos.org%2Fprivacy-policy&data=05%7C01%7Cvdia%40utk.edu%7C7f95ea4e9e3f4595a22808db34665e1f%7C515813d9717d45dd9eca9aa19c09d6f9%7C0%7C0%7C638161386490766846%7CUnknown%7CTWFpbGZsb3d8eyJWIjoiMC4wLjAwMDAiLCJQIjoiV2luMzIiLCJBTiI6Ik1haWwiLCJXVCI6Mn0%3D%7C3000%7C%7C%7C&sdata=ubYMTk%2BthPd9eQy%2FA%2BotdFAuXpxsCg1H3YfrnigSpks%3D&reserved=0).

Reviewer #1: No

Reviewer #3: No

**Response.** Thank you very much.
